# Physical activity and sedentary time among preschoolers in centre-based childcare: a systematic review

**DOI:** 10.1186/s12966-018-0745-6

**Published:** 2018-11-21

**Authors:** Kathleen T. O’Brien, Leigh M. Vanderloo, Brianne A. Bruijns, Stephanie Truelove, Patricia Tucker

**Affiliations:** 1 Department of French Studies, Faculty of Arts and Humanities, University of Western Ontario, London, Ontario Canada; 2 Child Health Evaluative Sciences, The Hospital for Sick Children, Toronto, Ontario Canada; 3 Health and Rehabilitation Sciences, Faculty of Health Sciences, University of Western Ontario, London, Ontario Canada; 4 School of Occupational Therapy, Faculty of Health Sciences, University of Western Ontario, 1201 Western Road, Elborn College, Room 2547, London, N6G 1H1 Ontario Canada

**Keywords:** preschoolers, physical activity, sedentary time, centre-based childcare, systematic review, accelerometry

## Abstract

**Background:**

Many preschoolers spend a substantial portion of their day enrolled in centre-based childcare; the amounts of physical activity and sedentary time accumulated in this environment are critical to preschoolers’ ability to meet movement guidelines. The purpose of this systematic review was to provide a comprehensive overview of the objectively assessed physical activity and sedentary time of preschoolers in centre-based childcare (registration no. CRD42016033502).

**Methods:**

Eight online databases were searched using terms related to physical activity, sedentary time, preschoolers and centre-based childcare. Published, peer-reviewed primary studies written in English that objectively assessed (via accelerometry) the physical activity and sedentary time of preschoolers (2-5 years) in centre-based childcare were included.

**Results:**

Fifty-five studies (published 2004-2017) from 11 countries, representing 13,956 participants were included. Studies reported light physical activity (*n*=38) ranging from 2.94 to 29.96 mins/hr, moderate-to-vigorous physical activity (*n*=46) which ranged from 1.29 to 22.66 mins/hr, and total physical activity (*n*=42) ranging from 4.23 to 47.17 mins/hr. Sedentary time (*n*=47) ranged from 12.38 to 55.77 mins/hr.

**Conclusion:**

Physical activity and sedentary time were highly varied and inconsistent between studies; therefore, it is difficult to determine preschoolers’ true amount of physical activity and sedentary time during childcare hours. Despite this variability, preschoolers were noted to participate in high rates of sedentary time in this setting. The lack of homogeneity is an important finding in and of itself as it highlights the lack of consistency in measuring, processing, and reporting paediatric physical activity data.

## Introduction

Levels of physical activity and sedentary time among young children have been widely examined and discussed in the literature [1–4]. To understand the degree to which this population’s activity levels are associated with health outcomes, many countries have established physical activity and sedentary behaviour guidelines for young children under 5 years [5–8]. Specific to Canada, the 24-Hour Movement Guidelines recommend participation in at least 180 minutes of physical activity per day for children aged 1-4, including 60 minutes of energetic play (moderate-to vigorous-physical activity [MVPA]) for those 3-4 years [7]. In Canada, Australia, and New Zealand screen-viewing should not exceed more than 60 min for children 2-4 years, and those under 2 should not engage in any screen use [7]. At 5 years of age, children should engage in 60 minutes of MVPA each day, and limit recreational screen-viewing to 120 min per day [8]. These guidelines provide benchmarks for parents, public health representatives, and early childhood educators to strive to provide opportunities and support for young children to meet these recommendations.

In an effort to improve the activity levels of young children, the childcare environment may be a worthwhile setting to intervene – many children are enrolled in these programs and spend a large proportion of their time therein [9, 10]. In examining this environment, a large Canadian-based study of preschoolers (*n*=297) found that in comparison to those attending full-day kindergarten and home-based childcare, young children enrolled in centre-based care spent the most time being sedentary at 41.62 mins/hr [2]. Similarly, Vanderloo et al. (2014) reported in their Canadian study that preschoolers in centre-based childcare (*n=*71*)* only spent 1.58 mins/hr in MVPA [11]. In contrast, Mazzucca et al. (2017) reported that children engaged in 55 minutes of MVPA per childcare day, and that physical activity levels varied between indoor and outdoor activities – a substantial variation from the results produced by Vanderloo and colleagues [12]. Young children have been reported to spend a considerable amount of time in childcare, [10] and in these venues, the rates of physical activity have been documented to be low and sedentary time high; therefore, centre-based childcare represents an ideal setting to foster participation in the recommended amount of physical activity, while undertaking efforts to discourage excessive sedentary time [13–17].

A systematic review of objectively measured physical activity and sedentary time (accelerometers only – the gold standard for this population [18]) of preschoolers’ waking hours has been conducted [3]. Specifically, Hnatiuk et al. (2014) reported that the proportion of time spent in these behaviours varied greatly, ranging from 2 to 41% for MVPA and from 34 to 94% for sedentary time, as a result of differences in study design and methods of data processing [3]. Consequently, a clearer picture of young children’s activity behaviours is needed, specifically in environments like childcare, where centre characteristics have been noted to be a strong influence on these behaviours [1, 19]. While a recent review by Vanderloo, Martyniuk, and Tucker (2015) explored both physical activity and sedentary time of preschoolers in home-based childcare facilities, [20] no systematic review has looked at objectively measured physical activity and sedentary time of preschoolers in centre-based childcare. With research pertaining to the centre-based childcare environment rapidly arising in physical activity literature, as well as recent improvements in activity measurement protocols, it is timely to undertake a synthesis of this work to direct future research efforts and interventions in this setting, as well as government policy. Therefore, the purpose of this study was to systematically review preschoolers’ physical activity and sedentary time during centre-based childcare hours, as measured by accelerometry.

## Methods

This review was registered with PROSPERO (no. CRD42016033502), and adheres to the PRIMSA statement for systematic reviews [21, 22].

### Search Strategy

In consultation with a Health Sciences Librarian, a comprehensive search strategy was developed and used to explore young children’s physical activity and sedentary time during centre-based childcare hours. Eight electronic databases were searched: CINAHL, Medline, ProQuest, PsychInfo, EMBASE, Scopus, Sport Discus, and Physical Education Index. Search terms focused on physical activity, sedentary time, preschoolers, and centre-based childcare (see Table 1 for a sample search strategy). Database searches ceased on February 10, 2017. Manual searches of four journals’ (i.e., *Pediatric Exercise Science*, *Medicine and Science in Sports and Exercise*, *Journal of Physical Activity and Health*, and *International Journal of Behavioral Nutrition and Physical Activity*) “in press” or “ahead of print” sections, as well as the reference lists of included studies, were reviewed to ensure a thorough and comprehensive search was undertaken. International experts in the field of interest were also contacted to ensure all appropriate literature was captured. The search results were exported and saved in Mendeley (version 1.17.9; referencing software), where duplicates were manually deleted to establish a complete list of articles for screening.

**Table 1:** Sample Search Strategy (EMBASE)

#	Search Term	Results	Search Type
1	preschool child/	547180	Advanced
2	“preschoolers”.mp	5836	Advanced
3	toddler.mp.	7394	Advanced
4	toddlers.mp.	7518	Advanced
5	“early years”.mp.	4177	Advanced
6	“early childhood”.mp.	29039	Advanced
7	“preschool-aged child”.mp.	47	Advanced
8	“preschool-aged children”.mp.	1616	Advanced
9	“young child”.mp.	4684	Advanced
10	“young children”.mp.	51507	Advanced
11	1 OR 2 OR 3 OR 4 OR 5 OR 6 OR 7 OR 8 OR 9 OR 10	1459133	Advanced
12	childcare.mp OR exp child care/	64155	Advanced
13	“childcare centre”.mp.	31	Advanced
14	“centre based childcare”.mp.	17	Advanced
15	“center based childcare”.mp.	18	Advanced
16	“center-based childcare”.mp.	18	Advanced
17	“centre-based childcare”mp.	17	Advanced
18	“day care”.mp.	14895	Advanced
19	“early learning centre”.mp.	1	Advanced
20	“early learning center”.mp.	2	Advanced
21	physical activity.mp. OR physical activity/	155492	Advanced
22	exercise.mp.	433622	Advanced
23	movement.mp.	361251	Advanced
24	“active play”.mp.	220	Advanced
25	“locomotor activity”.mp.	24357	Advanced
26	“motor activity”.mp.	54511	Advanced
27	“physical exertion”.mp.	2798	Advanced
28	“active movement”.mp.	1274	Advanced
29	“outdoor play”.mp.	217	Advanced
30	outdoor time.mp.	250	Advanced
31	recess.mp.	115	Advanced
32	“sedentary behaviour”.mp.	1547	Advanced
33	sedentary lifestyle/ or sedentary.mp	34250	Advanced
34	Inactive.mp.	106149	Advanced
35	stationary.mp.	57673	Advanced
36	“physical inactivity”.mp.	9087	Advanced
37	“sedentary activity”.mp.	597	Advanced
38	12 OR 13 OR 14 OR 15 OR 16 OR 17 OR 18 OR 19 OR 20	170150	Advanced
39	21 OR 22 OR 23 OR 23 OR 24 OR 25 OR 26 OR 27 OR 28 OR 29 OR 30 OR 31 OR 32 OR 33 OR 34 OR 35 OR 36 OR 37	1593829	Advanced
40	11 AND 38 AND 39	802	Advanced

Note: This table was originally published in a review by Truelove et al. [51] and has been reproduced here

### Study Eligibility

Study eligibility criteria included: 1) primary studies; 2) written in English; 3) published in a peer-reviewed journal; 4) healthy (i.e., free from chronic diseases or developmental delays) preschool children (2 to 5 years) enrolled in centre-based childcare; 5) physical activity and/or sedentary time during centre-based childcare hours measured via accelerometry; and, 6) physical activity and/or sedentary time measured for at least 3 hours on one or more days.

### Screening for Inclusion

The titles and abstracts of all studies captured from the database searches were reviewed independently by two researchers. To ensure that all eligibility criteria were considered, reviewers used a screening form developed by the research team, and adapted from previous studies [16, 20]. The reviewers discussed conflicting views on the eligibility of an article, and a third researcher was consulted when necessary. All articles that were deemed eligible for inclusion were subsequently reviewed in their full-text form. In instances where the full-text article could not be retrieved via the university’s library repository, authors were emailed directly by the research team. Three researchers independently read each article in its entirety, utilizing a full-text screening form designed in advance. Discrepancies in decisions to include or exclude, as well as any concerns regarding eligibility were directed to a fourth researcher.

### Extraction of Data

After the final list of included articles was established, all relevant data were extracted. The information collected via the extraction table included authors, the year of publication, the country in which the study was conducted, information regarding the sample (e.g., size, age range), the model of accelerometer used (e.g., Actical, ActiGraph, etc.), data processing decisions (e.g., wear time, epoch length, cut-points applied, etc.) and levels of physical activity (light, MVPA, total) and sedentary time reported (e.g., mins/hr, % of time, etc.).

### Quality Assessment

The quality of the studies was assessed using the checklist proposed by Downs and Black [23]. All articles were assessed by two reviewers, with a third reviewer serving as an arbitrator, if necessary. For randomized controlled trials, the full checklist (27 questions) was used. A modified version of the checklist (10 questions) was applied to all other study types, and is in-line with previous research studies [24, 25]. The quality score of each article can be found in Table 2. Although not established a priori, all studies included were of high quality (i.e., scored from 21-30 using the full checklist, or 7-10 using the modified checklist) [23, 24].

**Table 2:** Study Characteristics for Included Studies separated by Accelerometer Type (n=55)

Authors (Year)	Country	Study Design	Sample Size & Age (yrs)	Average Wear-Time	Monitoring Time During Childcare	Epoch Length	Cut-Points	Mean Physical Activity (mins/hr)	Mean Sedentary Time (mins/hr)	Study Quality
Carson et al. (2015a) [33]	Canada	Cohort	N= 50 Age= 3-5	5.6 hr/day (SE= 0.2)	5 days of monitoring; ≥1 hr of wear-time on ≥3 days to be included	15-s	Adolph	LPA= 19.50 MVPA= 5.30 TPA= 24.80^b^	35.20	9^c^
Chow et al. (2016) [35]	Canada	Longitudinal	N= 69 Age= 3-5	11.4 hr/day	7 days of monitoring; ≥8 hr of wear-time on ≥1 day to be included	15-s	Puyau	LPA = 21.30^b^ MVPA = 4.40^b^ TPA = 26.00^b^	33.48^b^	7^c^
Copeland et al. (2016) [29]	U.S.	Cross-sectional	N= 388 Age= 4.3	8.4 hr (SD=1.2)	1 day of monitoring	15-s	Pfeiffer	LPA = 21.40^a^ MVPA= 2.40^a^ TPA = 23.80^ba^	36.10	10^c^
Goldfield et al. (2016) [52]	Canada	Cluster RCT	N= 83 Age= 3-5	6.7 hr/day	5 days of monitoring; ≥4hr of wear-time on ≥2 days to be included	15-s	Adolph	LPA= 18.51^a^ MVPA= 4.90^a^ TPA= 23.43^a^	36.85	27^d^
Tucker et al. (2015) [2]	Canada	Cross-sectional	N= 218 Age= 2.5-5	6.8 hr/day	5 days of monitoring; ≥5 hr of wear-time on ≥3 days to be included	15-s	Pfeiffer	Not reported	41.62 (SD=3.78)	10^c^
Van Cauwenberghe et al. (2012b) [38]	New Zealand	Validation	N= 49 Age= 3-4	4.9 hr/day	1 day of monitoring; ≥3 hrs of concurrent data on both devices to be included	15-s	Evenson	Not reported	30.61^b^	9^c^
Vanderloo & Tucker (2015) [32]	Canada	Cross-sectional	N= 101 Age= 2.5-5	5.9 hr/day	5 days of monitoring; ≥5 hr of wear-time of ≥1 day to be included	15-s	Pfeiffer	LPA=16.42 MVPA= 1.37 TPA= 17.70	42.38	10^c^
Vanderloo et al. (2015) [11]	Canada	Cross-sectional	N= 71 Age= 2.5-5	6.8 hr/day	5 days of monitoring; ≥5 hr of wear-time on ≥3 days to be included	15-s	Pfeiffer	LPA = 16.78^b^ MVPA= 1.58 TPA= 18.36	Not reported	10^c^
Vanderloo et al. (2014) [19]	Canada	Cross-sectional	N= 31 Age= 4.10	7.5 hr/day	1 day of monitoring; ≥4hr of wear-time to be included	15-s	Pfeiffer	LPA = 15.88 MVPA= 1.54 TPA= 17.42	40.64	10^c^
Webster et al. (2015) [26]	U.S.	Cross-sectional	N = 118 Age= 2.5-5	4.3 hr/day	4 days of monitoring	15-s	Pfeiffer	MVPA = 1.77^b^	Not reported	8^c^
ActiGraph										
Addy et al. (2014) [53]	U.S.	Cross-sectional	In-School Sample: N=199 Age 4.2	8.2 hr/day (SD=1.4)	7 days of monitoring; ≥4.9hr of wear-time on ≥4 days to be included	15-s	Pate	LPA = 5.70 MVPA= 7.50 TPA= 13.20	46.80^b^	9^c^
Alhassan et al. (2007) [27]	U.S.	RCT	N= 32 Age= 3-5	13.0 hr/day	4 full days of monitoring; School day: 8:00 a.m. to 5:00 p.m.	30-s epoch, converted to 60-s	Sirard	LPA = 2.94^a^ MVPA = 1.29^a^ TPA = 4.23^a^	55.77^b^	25^d^
Alhassan et al. (2012a) [54]	U.S.	Cluster RCT (pilot study)	N= 71 Age= 2.9-5	9.2 hr/day	7 days of monitoring; ≥9hr of wear-time on ≥4 days to be included	15-s	Sirard	LPA = 9.90^a^ MVPA = 4.32^a^ TPA = 14.22^a^	45.75^a^	25^d^
Alhassan et al. (2012b) [55]	U.S.	Cluster RCT	N= 291 Age= 2.9-5	Not reported	5 days of monitoring; School day: 7:00am-4:30pm	15-s	Sirard	LPA= 11.16^b^ MVPA = 3.84^b^ TPA = 15.00^b^	44.52^b^	25^d^
Alhassan et al. (2013) [56]	U.S.	Cluster RCT (pilot study)	N= 67 Age= 2.9-5	12.2 hr/day	≥9 hr of wear-time on ≥4 days to be included	15-s	Sirard	LPA = 9.99^a^ MVPA = 4.08^a^ TPA = 14.07^a^	46.14^a^	28^d^
Alhassan et al. (2016) [57]	U.S.	Cluster RCT	N= 291 Age= 4.1	6.9 hr/day	≥7.0 hrs of wear-time ≥3 days to be included	15-s epochs, converted to 60-s	Sirard	LPA= 11.55^a^ MVPA = 3.96^a^ TPA= 15.51^a^	44.76^a^	23^d^
Annesi et al. (2013a) [58]	U.S.	Cluster RCT	N= 275 Age= 3.5-5.6	4.8 hr/day	School day: 9:15 a.m. to 2:00 p.m.	15-s	Pate	LPA= 11.44^a^ MVPA= 18.64^a^ TPA= 30.08^a^	29.92^a^	23^d^
Annesi et al. (2013b) [59]	U.S.	Cluster RCT	N= 885 Age= 3-5	4.8 hr/day	School day: 9:15 a.m. to 2:00 p.m.	15-s	Pate	LPA = 10.40^a^ MVPA= 13.92^a^ TPA = 24.33^a^	35.67^a^	24^d^
Annesi et al. (2013c) [34]	U.S.	RCT	N= 338 Age= 3-5	4.8 hr/day	School day: 9:15 a.m. to 2:00 p.m.	15-s	PA : Pate ST : Sirard	LPA=11.59^a^ MVPA= 19.16^a^ TPA= 30.75^a^	29.26^a^	24^d^
Barbosa et al. (2016) [28]	Brazil	Cross-sectional	N= 370 Age= 5.2	Not reported	5 days of monitoring; ≥6 hr of wear-time to be included	1-s	Sirard	LPA= 4.38 MVPA= 1.40 TPA= 5.85	54.15	9^c^
Byun et al. (2013) [60]	U.S.	Cross-sectional	N= 331 Age= 4	5.9 hr/day	5 days of monitoring; ≥4 hr of wear-time on ≥3 days to be included	15-s	Pate	Not reported	45.75	8^c^
Carson et al. (2015b) [61]	Australia	Cohort	N= 177 Age= 3-5	4.4 hr/day	7 days of monitoring; ≥6 hr of wear-time on ≥3 days to be included	15-s	Evenson	Not reported	28.68^b^	9^c^
Cerin et al. (2016) [62]	Australia	Observational	N= 84 Age= 3-5	11.7 hr/day	7 days of monitoring; ≥8 hours of wear-time on ≥1 day to be included	15-s, converted to 30-s	Pate	LPA= 22.40^b^ MVPA= 4.20^b^ TPA= 29.40^b^	30.60^b^	10^c^
Dawson-Hahn et al. (2015) [63]	U.S.	Cross-sectional	N= 81 Age= 3-5	11.7 hr/day	≥3 hr of wear-time on ≥5 days to be included	15-s	Pate	TPA= 24.90	35.10	7^c^
De Craemer et al. (2014) [36]	Belgium	Cluster RCT	N= 472 Age= 4-6	11.8 hr/day (SD=1.1)	6 days of monitoring; ≥6 hr of wear-time on ≥2 weekdays and ≥1 weekend day	15-s	Evenson	LPA= 29.04^a^ MVPA= 4.08^a^ TPA= 33.48^a^	26.52^a^	27^d^
De Craemer et al. (2016) [64]	Belgium	Cluster RCT	N= 859 Age= 3-4	11.8 hr/day (SD=1.1)	6 days of monitoring; ≥6 hr of wear-time on ≥2 weekdays and ≥1 weekend day	15-s	Evenson	TPA= 32.82^a^	27.18^a^	26^d^
Erinosho et al. (2016) [65]	U.S.	Cross-sectional	N= 544 Age= 3-5	6.8 hr/day (SD=1.3)	4 days of monitoring; ≥4 hr of wear-time on 4 days to be included	5-s converted to 15-s	ST: Evenson, PA: Pate	LPA = 23.4^b^ MVPA= 5.5 TPA = 28.90^b^	31.1	9^c^
Gagné & Harnois (2013) [66]	Canada	Cross-sectional	N= 242 Age= 3-5	8.0 hr/day	4 days of monitoring; ≥4 hr of wear-time on ≥2 days to be included	15-s	Sirard	LPA = 5.05^b^ MVPA = 1.58^b^ TPA = 6.63^b^	53.37^b^	10^c^
Henderson et al. (2015) [30]	U.S.	Cross-sectional	N= 389 Age= 3-5	3.33 hr (SD=0.7)	1 day of monitoring	5-s	Evenson	LPA= 29.96^b^ MVPA= 9.00 TPA = 30.96^b^	29.82^b^	9^c^
Hinkley et al. (2016) [67]	Australia	Observational	N= 731 Age= 3-5	Not reported	8 days of monitoring; ≥50% of preschool time on ≥2 days to be included	15s	Troiano	TPA= 29.61^a^	30.39^a^	10^c^
Jones et al. (2011) [68]	Australia	RCT	N= 150 Age= 4	Not reported	2 days of monitoring	15-s	Sirard	LPA= 21.91^a^ MVPA= 10.10^a^ TPA= 32.01^a^	27.99^a^	25^d^
Loprinzi & Trost (2010) [69]	Australia	Cross-sectional	N= 156 Age= 2-5	5.5 hr/day (SD= 0.5)	5 days of monitoring; ≥4 hr of wear-time on ≥2 days to be included	15-s	Sirard	MVPA= 9.10	Not reported	8^c^
Oleson et al. (2013) [70]	Denmark	Cluster RCT	N= 426 Age= 5.8	7.2 hr/day	5 days of monitoring; ≥3 hr of wear-time on ≥3 days to be included;	15-s	Evenson	MVPA= 8.16^b^	Not reported	25^d^
O'Dwyer et al. (2014) [71]	UK	Cross-sectional	N= 188 Age= 4-5	6 hr/day	7 days of monitoring; ≥10.3 hr of wear-time during a weekday on ≥2 weekdays School day: 9:00 a.m. to 3 p.m.	5-s	Sirard	MVPA= 4.06^ba^	Not reported	10^c^
O'Neill et al. (2016) [72]	U.S.	Cross-sectional	N=341 Age= 3-5	5.5 hr/day (SD=1.5)	5 days of monitoring; ≥6 hr of wear-time on ≥2 days to be included	15-s	ST: España-Romero, PA: Pate	LPA= 7.00^b^ MVPA= 7.10 TPA= 14.10	45.90^b^	7^c^
Pagels et al. (2011) [73]	U.S. and Sweden	Cross-sectional	N= 55 Age= 3.4-5.7	7.2 hr/day (SD=1.2)	5 days of monitoring	15-s	Sirard	MVPA= 2.36^a^ TPA= 6.65^a^	54.92^a^	9^c^
Pate et al. (2016) [74]	U.S.	RCT	N= 379 Age= 3-5	5.3 hr/day	5 days of monitoring; ≥3 days to be included	15-s	Pate	LPA= 7.00^a^ MVPA= 7.45 ^a^ TPA= 13.90^a^	46.10^a^	26^d^
Pate et al. (2014) [75]	U.S.	Cross-sectional	N= 301 Age= 4	5.8 hr/day	5 days of monitoring; ≥4 hr of wear-time on ≥3 days to be included	15-s	ST: España-Romero PA: Pate	LPA= 7.10^a^ MVPA= 7.10^a^ TPA= 14.20^a^	45.80^b^	10^c^
Pate et al. (2004) [1]	U.S.	Cross-sectional	N= 247 Age= 3-5	4.4 hr/day (SD= 1.3)	1 to 11 days of monitoring; ≥1 hr of wear-time on ≥3 days to be included	15-s	Sirard	LPA= 10.50^a^ MVPA= 7.40^a^ TPA= 17.90^ba^	42.10	10^c^
Ross et al. (2013) [76]	U.S.	Cross-sectional	N= 339 Age= 3-5	Not Reported	5 days of monitoring	15-s	Pate	LPA = 7.55^a^ MVPA= 7.75^a^ TPA= 15.30^a^	44.70^b^	26^d^
Saunders et al. (2017) [77]	U.S.	Cross-sectional	N= 567 Age= 3-5	Not Reported	5 days of monitoring; ≥50 % of time in childcare on ≥3 days to be included	15-s	Pate	MVPA=6.80	Not reported	10^c^
Schlechter et al. (2017) [78]	U.S.	Cross-sectional	N= 73 Age= 3-6	6.4 hr/day	15 days of monitoring	15-s	Van Cauwenberghe	TPA = 18.30^b^	41.70^b^	10^c^
Schuna et al. (2016) [41]	U.S.	Cross-sectional	N= 62 Age= 3-5	5.2 hr/day	5 days of monitoring at 2 separate time points (fall and winter); ≥3 hr of wear-time on ≥2 days to be included	5-s	Pate	LPA= 16.20^a^ MVPA= 8.45^a^ MPA= 5.25^a^	35.35^b^	8^c^
Shen et al. (2013) [79]	U.S.	Cross-sectional	N= 46 Age= 3-5	6.8 hr/day	6 days of monitoring; Data collected from: 7am to 8pm	15-s	Sirard	LPA= 10.23^a^ MVPA= 3.58^a^	46.20^b^	9^c^
Soini et al. (2014) [40]	Finland/Australia	Cross-sectional	N= 121 Age= 3	10.2 hr/day	5 days of monitoring; ≥7.5 hr of wear-time on ≥1 childcare day and ≥1 homecare day	5-s	Pate	LPA= 18.00^ba^ MVPA= 8.40^ba^ TPA= 26.40^ba^	33.60^b^	10^c^
Stephens et al. (2014) [80]	U.S.	Cross-sectional	N= 1,352 Age= 2-5	Not reported	1 or 2 days of monitoring	15-s, (some children w/ 60-s due to malfunction)	Pate	MVPA= 6.20^a^	Not reported	9^c^
Sugiyama et al. (2012) [81]	Australia	Cross-sectional	N= 89 Age= 3-5	6.6 hr/day	3 days of monitoring	15-s	Sirard	LPA = 7.56^b^ MVPA= 3.67^b^ TPA = 11.23^b^	48.77^b^	9^c^
Tandon et al. (2015) [82]	U.S.	Cross-sectional	N= 98 Age= 3-5	Not reported	4 days of monitoring; School day: 9:00 a.m. to 5:00 p.m.	15-s	Pate	LPA = 7.80^b^ MVPA= 8.40^b^ TPA = 16.20^b^	43.80^b^	10^c^
Vale et al. (2011) [83]	Portugal	Cross-sectional	N= 59 Age= 2-5	Not reported	4 days of monitoring	5-s	Sirard	LPA = 6.09^b^ MVPA = 4.52^b^ TPA = 10.61^b^	49.39^b^	7^c^
Vale et al. (2009) [84]	Portugal	Cross-sectional	N= 59 Age= 2-5	Not reported	5 days of monitoring; ≥6 hours of wear-time to be included	5-s & 60-s	Sirard	5-s epoch = MVPA = 26.46 (SD= 9.64) 60-s epoch = MVPA = 10.05 (SD= 8.43)	Not reported	7^c^
Van Cauwenberghe et al. (2012a) [85]	Belgium	Cross-sectional (pilot study)	N= 107 Age= 4-6	7.7 hr/day	5 days of monitoring	15-s	Van Cauwenberghe	LPA = 4.26^b^ MVPA = 5.25^b^ TPA = 9.51^b^	50.49^b^	7^c^
Van Cauwenberghe et al. (2013) [86]	Belgium	Cross-sectional	N= 200 Age= 4-6.2	12.2 hr/day	4 days of monitoring; ≥8 hours of wear-time on ≥2 days to be included	15-s	Van Cauwenberghe	LPA= 4.20^b^ MVPA= 4.31^b^ TPA= 8.51^b^	51.49^b^	9^c^
Williams et al. (2008) [87]	U.S.	Cross-sectional	N= 198 Age= 3-4	12.7 hr/day	5 days of monitoring	15-s	Pate	LPA= 19.56^b^ MVPA= 7.56^b^ TPA= 27.12^b^	32.88^b^	8^c^
Actiheart										
Hesketh et al. (2015) [31]	UK	Cross-sectional	N= 202 Age= 3-4	10.8 hr/day	7 days of monitoring;≥10 hr of wear-time on ≥2 days to be included	15-s	Pate	LPA= 25.49 MVPA= 22.66 TPA= 47.15	12.38	10^c^
ActivPAL										
Ellis et al. (2016) [37]	Australia	Cross-sectional	N= 233 Age= 3.0-5.9	5.1 hr/day	5 days of monitoring; ≥3 hr of wear-time on ≥1 day to be included	15-s	Not applicable	TPA = 10.98	49.02	10^c^
Van Cauwenberghe et al. (2012b) [38]	New Zealand	Validation	N= 49 Age= 3-4	4.9 hr/day	1 day of monitoring; ≥3 hrs of concurrent data on both devices to be included	15-s	Not applicable	Not reported	30.24	9^c^

*Notes*: *LPA* light physical activity, *MVPA* moderate-to vigorous-intensity physical activity, *TPA* total physical activity, *SD* standard deviation, *RCT* randomized control trial.

^a^ = averaged scores generated by researchers (e.g., scores between control and intervention group, scores between location, etc.).

^b^ = calculated activity intensity based on data presented in original article (i.e., converted mins/day to mins/hr at each activity intensity based on wear-time during childcare; converted % of time to mins/hr at each activity intensity; summed LPA and MVPA to generate TPA score).

^c^ = scored using the modified Downs & Black Checklist (out of 10 items)

^d^ = scored using the complete Downs & Black Checklist (out of 27 items)

### Data Synthesis and Analysis

To facilitate data synthesis, all included studies were separated into distinct categories depending on the model of accelerometer used. In all instances, the mean hourly rate of physical activity (light physical activity [LPA], MVPA, total physical activity [TPA]) and sedentary time was used to allow for easy comparison between studies. If data for particular intensity levels were not provided, simple calculations were carried out on the basis of available data in the paper. For example, if TPA was not reported, authors summed time spent in LPA and MVPA to derive this final number. For each accelerometer model, the mean for each intensity level across studies was calculated. Data for each level of activity intensity were grouped and synthesized into ranges. Meta-analyses were unable to be conducted given the heterogeneity of the reported study outcomes.

## Results

### Database Searches

**Fig. 1:**
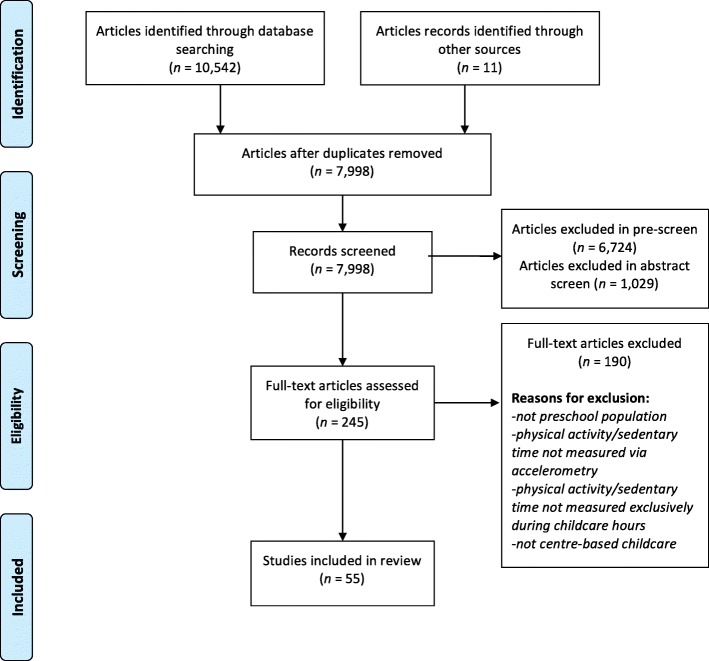
PRISMA Flow Diagram for Systematic Review

After searching eight electronic databases, 10,542 articles were captured and uploaded into Mendeley. An additional eight articles were retrieved after reviewing the reference lists of included studies, and three via the ‘ahead of print’ sections from four online journals, bringing the total number of articles to 10,553. Following the removal of duplicates and pre-screening for articles that did not focus on healthy young children, 1,274 articles underwent title and abstract screening by two independent reviewers. Subsequently, full-text review was completed for the remaining 245 articles, with 190 being excluded, leaving 55 articles to be included in the review. See Fig. 1 for the PRISMA flow diagram.

### Characteristics of Included Studies

Included studies were conducted in 11 countries (United States [*n=*28], Canada [*n=*8], Australia [*n=*8], United Kingdom [*n=*2], Belgium [*n=*4], Denmark [*n=*1], Portugal [*n=*2], New Zealand [*n=*2], Sweden [*n=*1], Brazil [*n=*1] and Finland [*n=*1]), with one study conducted in both the United States and Sweden, and another study in both Finland and Australia. The sample size of included articles ranged from 31 to 1,352, with a mean sample size of 254 young children; the 55 included studies represented a total of 13,956 participants. Date of publication ranged from 2004 to 2017, with the majority of articles (*n=32*, 58.2%) published between 2014-2017. A variety of study designs were employed, the most common being cross-sectional [*n=*35] and randomized controlled trial [*n=*14]. Both physical activity and sedentary time were measured in 43 of the studies, while the remaining 12 studies measured physical activity (*n*=8) or sedentary time (*n*=4) only. A summary of the characteristics of included studies can be found in Table 3.

**Table 3:** Summary of Characteristics for Included Studies

	# of studies
Years of publication (range)	2004-2017
Sample size (*# of participants per study* )	31 - 1,352
Country	
United States	28
Canada	8
Australia	8
United Kingdom	2
Belgium	4
Denmark	1
Portugal	2
New Zealand	2
Sweden	1
Brazil	1
Finland	1
Study Design	
Cross-sectional	35
Randomized controlled trial	14
Cohort	2
Longitudinal	1
Validation	2
Observational	2
Accelerometer model	
Actical	9
Actiheart	1
ActivPAL	2
ActiGraph	44
Cut-points used	
Pate et al.	19
Sirard et al.	17
Pfeiffer et al.	6
Evenson et al.	7
Van Cauwenberghe et al.	3

A variety of accelerometer models were used (Actical [*n=*9], Actiheart [*n=*1], and ActivPAL [*n=*2]); however, ActiGraph was by far the most common [*n=*44]. Average accelerometer wear-time ranged from 4.3 [26] to 13.0 [27] hrs/day with monitoring ranging from 1 to 11 days. Most studies used a 15-s epoch, 1[28], with observations as low as 1-s and 5-s. A variety of cut-points were applied in the various studies with the most frequently adopted including Pate et al. [*n=*19], Sirard et al. [*n=*17], Pfeiffer et al. [*n=*6], Evenson et al. [*n=*7], and Van Cauwenberghe et al. [*n=*3]. See Table 2 for complete study characteristics and outcome data.

### Physical Activity Prevalence Rates (LPA, MVPA, TPA)

Young children’s LPA ranged from: 15.88 [19] to 21.53 [29] mins/hr (*M* = 18.56) for Actical accelerometers; and 2.94 [27] to 29.96 [30] mins/hr (*M*= 11.80) for ActiGraph accelerometers. The one study which used Actiheart accelerometers reported 25.49 mins/hr of LPA. [31] LPA was not reported for the studies which used ActivPAL devices.

Rates of MVPA were lower for all accelerometer types, ranging from: 1.37 [32] to 5.30 [33] mins/hr (*M* = 2.91) for Actical accelerometers; and 1.29 [27] to 19.16 [34] mins/hr (*M*= 6.67) for ActiGraph accelerometers. The one study which used Actiheart accelerometers reported 22.66 [31] mins/hr of MVPA. MVPA was not reported for the two studies which used ActivPAL devices. While observed rates of MVPA were lower than LPA, the rates of MVPA measured via Actical were noticeably lower than those captured via ActiGraph (which produced a 17.87 mins/hr dispersion), with Actiheart accelerometer producing the highest MVPA score.

**Fig. 2:**
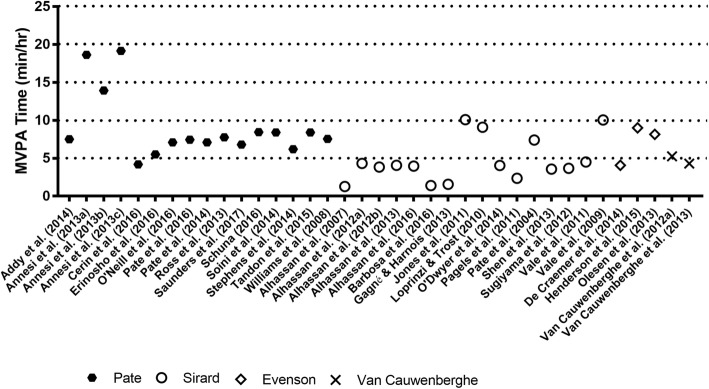
Time spent in MVPA based on device type (ActiGraph) and cut-points used

TPA accumulated by preschoolers in centre-based childcare ranged from 17.42 [19] to 26.00 [35] mins/hr (*M* = 21.64) for Actical accelerometers; and 4.23 [27] to 33.48 [36] mins/hr (*M*= 18.42) for ActiGraph accelerometers. TPA was 47.17 [31] mins/hr for the one study which used Actiheart accelerometers, and 10.98 [37] mins/hr for the study which used ActivPAL accelerometry. Figures 2 (ActiGraph) and 3 (Actical and Actiheart) illustrate the hourly rates of physical activity by applied cut-point. These figures illustrate that, generally speaking, studies that employed the same cut-points had hourly rates of MVPA that were comparable.

**Fig. 3:**
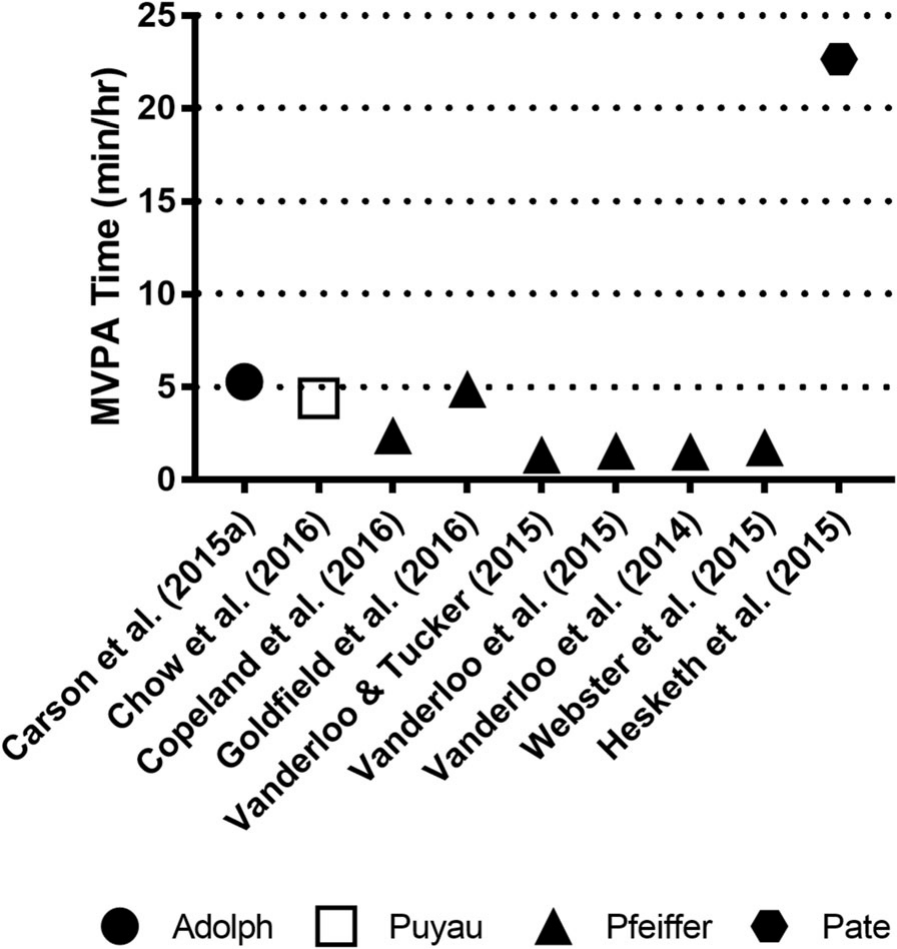
Time spent in MVPA based on device type (Actical and Actiheart) and cut-points used

As 11 countries are represented across this review, activity levels were also analyzed across four geographic regions: North America, South America, Europe and South Pacific (i.e. Australia and New Zealand). Of note, only one study originated from South America (Brazil). Mean LPA was very similar between North America and Europe (13.06 and 14.51 mins/hr, respectively); however, the preschoolers in the South Pacific region seemed to have accumulated more LPA (*M*= 17.46 mins/hr), while those in the South American study achieved much less (4.38[28]mins/hr). In terms of mean MVPA, the North American and South Pacific regions were quite comparable (6.13 and 7.09 mins/hr, respectively). Mean MVPA across the European studies was slightly higher at 9.12 mins/hr, while the South American study was quite low in comparison (1.40 [28] mins/hr). When considering the mean TPA across studies, preschoolers in the South Pacific region appear to be the most active (*M*= 23.27 mins/hr), followed by Europe (*M*=21.89 mins/hr), North America (*M*=19.57 mins/hr), and South America (5.85 [28] mins/hr).

### Sedentary Time Prevalence Rates

**Fig. 4:**
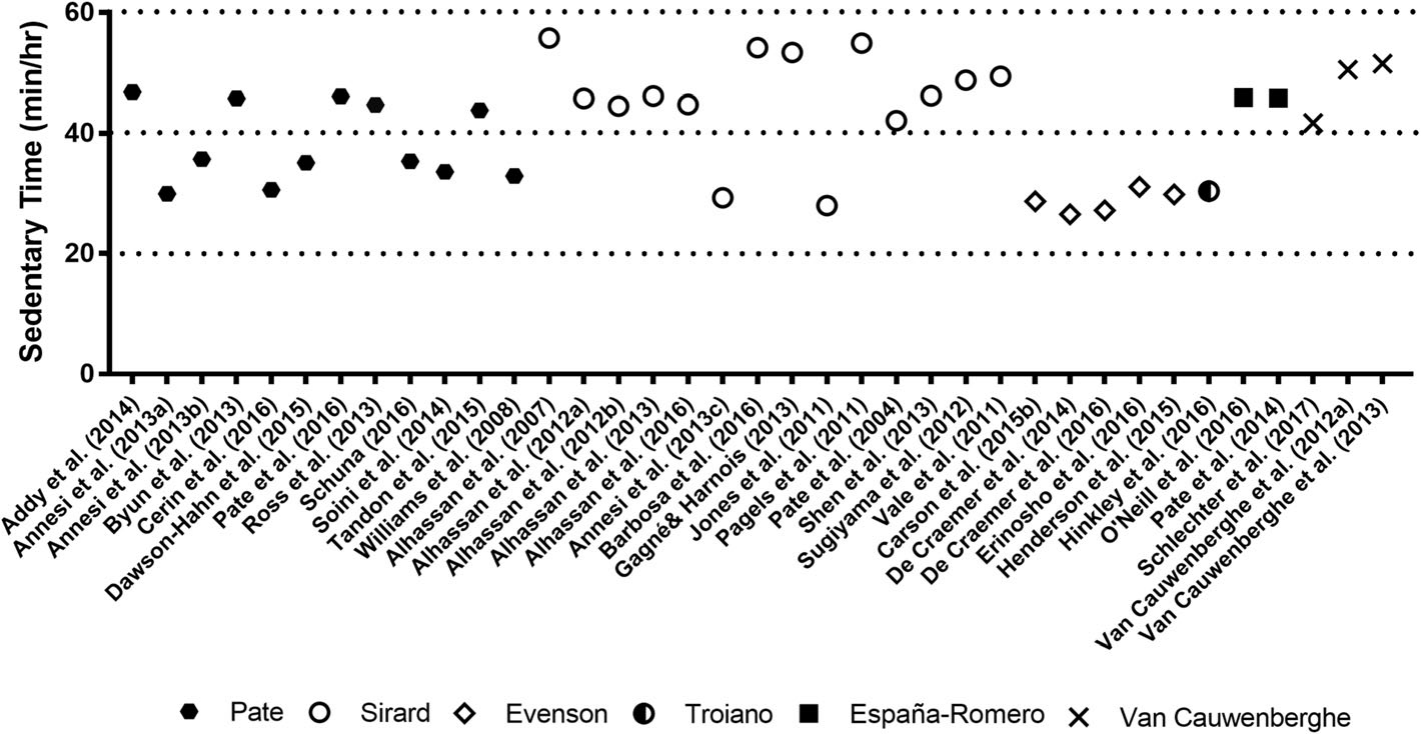
Time spent sedentary based on device type (ActiGraph) and cut-points used

Figures 4 (ActiGraph) and 5 (Actical and Actiheart) display the hourly rates of sedentary time by cut-point applied which illustrates that studies using the same cut-points cluster together. The hourly rates of sedentary time ranged from: 30.61 [38] to 42.38 [32] mins/hr (*M*= 36.47) for Actical accelerometers; 26.52 [36] to 55.77 [27] mins/hr (*M*= 40.88) for ActiGraph accelerometers; and 30.24 [38] to 49.02 [37] mins/hr (*M*= 39.63) for ActivPAL accelerometers. The one study that used Actiheart accelerometers reported 12.38 [31] mins/hr of sedentary time.

**Fig. 5:**
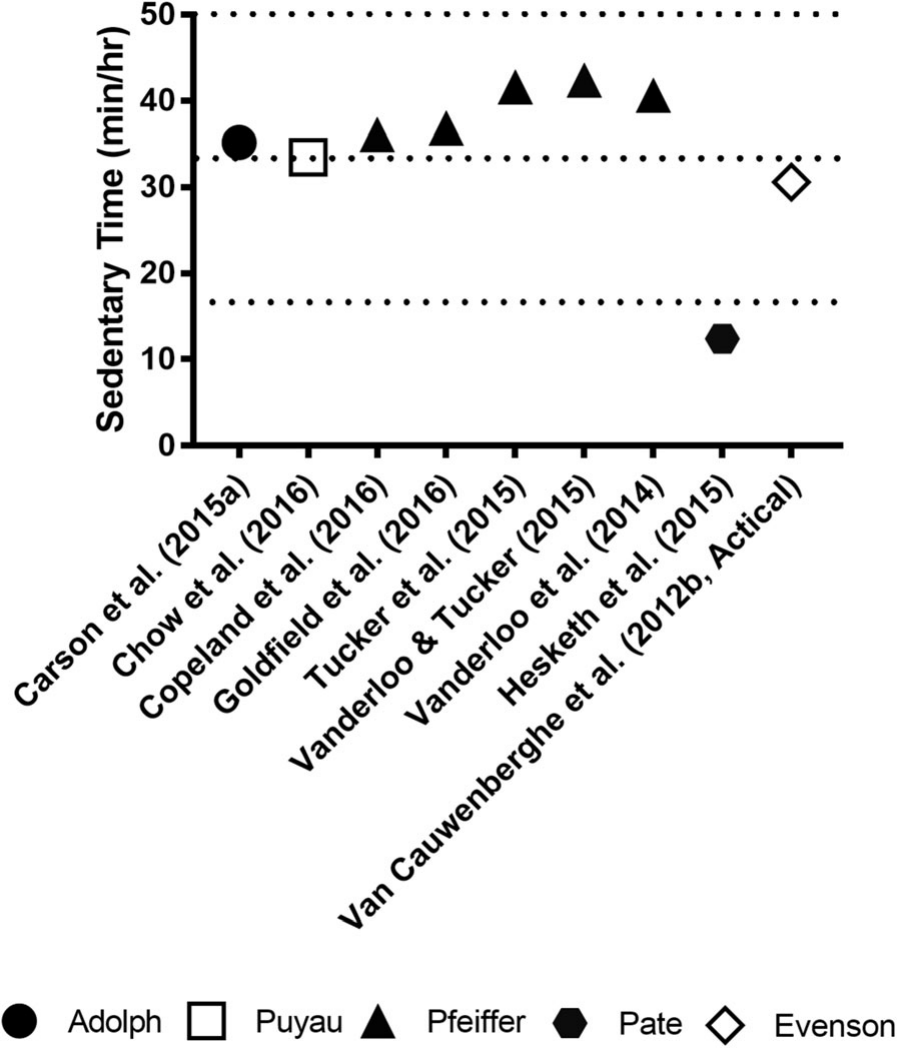
Time spent sedentary based on device type (Actical and Actiheart) and cut-points used

In exploring sedentary time by geographic region, North American preschoolers appeared to be much more sedentary than those from the Pacific region (41.23 versus 34.43 mins/hr). European studies reported a mean sedentary time that was intermediate to these two regions at 38.25 mins/hr. The sole South American study reported preschoolers’ sedentary time to be 54.15 [28] mins/hr.

## Discussion

The purpose of this systematic review was to gain a clearer picture of young children’s physical activity and sedentary time in centre-based childcare. This paper offers the first synthesis of objectively-measured physical activity and sedentary time in this unique setting among preschoolers. Given the dramatic increase in studies conducted in this environment and with this population, coupled with the ongoing challenges of comparing studies using different accelerometers, this study provides a snapshot of current rates of physical activity and sedentary time captured in centre-based childcare.

The results are highly variable, reporting that physical activity in childcare ranged from 2.94 [27] to 29.96 [30] mins/hr for LPA; 1.29 [27] to 22.66 [31] mins/hr for MVPA; and, 4.23 [27] to 47.17 [31] mins/hr for TPA, respectively. There are a number of reasons why these wide ranges were observed, namely, the different accelerometer devices and the cut-points used. The challenge of gathering an accurate picture of activity levels in centre-based childcare is also compounded by the diversity in data collection protocols (i.e., wear time criteria; trying to gather a habitual daily activity level), the output data produced and interpreted; (i.e., mins/hr, counts/min, %/wear time), and the variability in childcare centres (which has been shown to be a strong predictor of physical activity levels in this population [39]). With this in mind, Finn and colleagues noted that 50% of the variation in physical activity levels is a consequence of the childcare environment itself [39]; therefore, the wide ranges observed could also be a reflection of the varying quality of centre-based childcare facilities. As such, all these factors could be contributing to the varied magnitude in physical activity levels, and similarly sedentary time, observed.

Of note, 11 countries were represented in this review. Given different cultural norms and childcare practices, it is possible that geographic region may also influence inter-childcare centre variation. For example, in comparing the mean TPA reported by studies from the South Pacific region (Australia and New Zealand) versus North America, there is over 3.5 mins/hr difference in TPA (23.27 versus 19.57 mins/hr). When extrapolating these hourly rates over a full childcare day, this difference in activity is substantial. Furthermore, Soini et al. assessed the physical activity levels of 3-year-olds in childcare in both Finland and Australia [40]. Although they did not find inter-country differences in preschoolers’ activity levels, they describe various facets of the childcare centres from each country, for example structure of a typical day in childcare and its governance. Soini and colleagues also noted the ranges in outdoor temperature that were experienced throughout their study [40]. In a review as large as this, it is important to bear in mind that differences in variable such as governance, childcare structure and environment across countries could have an impact on the variation in reported activity levels. Seasonal differences between the countries included in this review could also play a role in producing a wide range of reported activity levels, as temperature differences could affect access to outdoor playtime, which is strongly correlated with activity levels among young children [12, 41, 42].

To clarify the ongoing challenge of comparing physical activity data from different accelerometer models, a recent study explored the differences in physical activity and sedentary time among preschoolers when administering Actical and ActiGraph accelerometer protocols. Specifically, Vanderloo and colleagues found that these two widely used devices, although validated by the same research team and protocols, capture different activity levels at 15- and 60-s epoch lengths [43]. This group determined that the Actical accelerometer reported higher levels of sedentary time, while the ActiGraph device captured more physical activity [43]. Likewise, although Borghese and colleagues noted good agreement between Actical and ActiGraph accelerometers in assessing older children’s MVPA levels (9-11 years), they also recognized the need to exercise caution when comparing across devices as reported activity levels are highly contingent on data reduction protocol and cut-points used [44]. These findings are supported in the current study as a greater variety of cut-points were applied in ActiGraph-adopted studies, and the rates observed were much wider. In an effort to produce more comparably measured and analyzed data, and therefore, providing a more consistent representation of young children’s physical activity levels, a uniform protocol for processing accelerometry data is needed.

This review confirms that interpreting preschoolers’ actual physical activity levels is challenging. Though not specific to the childcare environment, similar issues with variability of results were noted by Hnatiuk et al. in their review of objectively-measured activity levels in preschoolers (as measured by accelerometers, heart rate monitoring, and direct observation) [3]. While the goal of their review was not to explore activity levels in a particular setting, like childcare, they too highlighted the measurement challenges discussed above as impeding their ability to generate a “true” consensus on physical activity levels and sedentary time of preschool-age children [3]. This lack of interpretability makes it very difficult to determine whether young children are accumulating adequate time in physical activity during childcare hours to aid them in meeting the 24-Hour Movement Guidelines adopted by many countries [5–7]. With TPA values in this review ranging from 4.23 [27] to 47.17 [31] mins/hr, and with the assumption that two-thirds of their waking hours are spent in this childcare environment, [45, 46] it is unclear whether preschoolers would attain recommended minutes of physical activity [7]. For example, using the lower limit, young children would acquire 33.84 minutes in TPA during childcare hours, while the upper end would engage in 377.12 minutes during that same timeframe. Given the 24-hour Movement Guidelines for the Early Years encourage 180 minutes of TPA per day, [7] many children would be far surpassing this expectation, while some are well below it. Estimating MVPA time in line with the guidelines (i.e., 60 minutes per day) is equally ominous as rates ranged from 11.12 to 181.29 minutes during the childcare day [8]. Bornstein and colleagues in their 2011 meta-analysis reported a rate of 42.8 minutes per day of MVPA [47]. While not specific to the childcare setting, they too highlight the need for careful consideration when interpreting physical activity levels among this population and that steps are warranted to unify accelerometer-generated physical activity data to inform unbiased and improved comparisons across studies. Though some may perceive this inability to synthesize a univariate finding from the published data as a limitation, the present authors view this as a reflection of the lack of homogeneity in measuring, processing, and reporting objective physical activity data in the literature, while also bearing in mind the impact of factors such variation in childcare centre characteristics [39].

Similar to physical activity, reported sedentary time was mixed, though much higher levels of this behaviour were registered across studies. Specifically, time spent being sedentary ranged from 12.38 [31] to 55.77 [27] mins/hr. These findings could likely be attributed to the fact that the childcare environment is oftentimes referred to as a sedentary and/or obesogenic setting. Past work [2, 19, 48] highlight opportunities for sedentary behaviours (e.g. access to screens in childcare, etc.) as key contributors to high levels of this deleterious health behaviour. Limited outdoor space for free play and gross motor movement, combined with safety and liability concerns, may also be contributing factors worth considering. The types of sedentary behaviour may differ between geographic regions; however, this review did not measure behaviour types, as the focus was time spent in this intensity. This finding is in line with a previous review conducted by our research team in home- or family-based childcare, [20] where the results also documented low levels of physical activity and high levels of sedentary time among preschoolers. While these wide ranges are again, inhibiting a true depiction of sedentary levels among preschoolers in childcare, what is apparent is the higher rates noted, especially compared to MVPA. This has been confirmed elsewhere – Ellis et al. (2017) reported that preschoolers in childcare spend 48.4% of their time sitting, while only 19.1% in physical activity [37].

### Strengths and Limitations

The study provides a comprehensive synthesis of all studies measuring physical activity and sedentary time in centre-based childcare settings. In addition to the sheer magnitude of literature included, the review has provided rates of different activity levels within the context of the primary objective measurement techniques within this population. Despite efforts to provide meaningful data regarding activity levels in childcare, via mean hourly rates separated by accelerometer brand and examined across intensities, the methodological variations adopted between studies produced wide discrepancies in activity data. As such, a primary limitation of this review is the inability to provide a consistent picture of young children’s physical activity levels in childcare due to discrepancies or inconsistencies in how such data is collected and reported across published work. However, as previously discussed, this lack of clarity regarding young children’s activity levels also serves as an important finding where additional work is needed to address this knowledge gap. Second, while all studies were considered “high quality” based on the Downs and Black tool, [23] this assessment could not account for accelerometry protocol approaches and subsequent reporting [49]. The adoption of consistent study protocols (i.e., use of similar accelerometers, cut-points, and data reduction techniques) could potentially allow for this conclusion to be determined, and represents an ongoing challenge in the physical activity literature. Heightened attention should be paid to ideal accelerometer wear time and time spent monitoring children during childcare, as this could affect study quality. It is difficult, for example, to compare results of a study that monitored participants for one day in childcare with 7 hours of accelerometer wear time against a study where preschoolers wore accelerometers for 5 hours each day, yet were monitored for a week. Third, only English articles were captured in this review, thus relevant studies published in other languages may have been missed. Lastly, numerous articles in this review did not report participants’ TPA within their results, and though it could be calculated when adequate information was provided, reporting TPA values are important given the target outlined by current international movement guidelines [6, 7, 50].

## Conclusion

It was difficult to ascertain a consistent representation of this population’s activity levels due to inconsistencies in measurement approaches used in the literature. Factors related to childcare centre characteristics, as well as the geographical locations where the studies were conducted may also have contributed to this variation. While recognizing the disparities across included studies, it did appear that sedentary time, in comparison to MVPA, was high. Consistent and appropriate accelerometry protocols are essential to gain insight into the levels of activity and inactivity in centre-based childcare, and to help gain an *accurate* picture of the proportion of children meeting (or not) the new international 24-Hour Movement Guidelines. Such information could also further enable the creation and support of appropriate policies for this environment and may help to create a healthier daily experience for preschoolers.

## Abbreviations

LPA: Light physical activity
MVPA: Moderate-to-vigorous physical activity
PRISMA: Preferred Reporting Items for Systematic Reviews and Meta-Analyses
RCT: Randomized controlled trial
TPA: Total physical activity
